# Larynx during exercise: the unexplored bottleneck of the airways

**DOI:** 10.1007/s00405-014-3159-3

**Published:** 2014-07-18

**Authors:** Ola Drange Røksund, John-Helge Heimdal, Jan Olofsson, Robert Christiaan Maat, Thomas Halvorsen

**Affiliations:** 1 Department of Pediatrics, Haukeland University Hospital, N-5021 Bergen, Norway; 2 Institute of Physiotherapy, Bergen University College, Bergen, Norway; 3 Department of Otolaryngology and Head and Neck Surgery, Haukeland University Hospital, Bergen, Norway; 4 Department of Clinical Medicine, University of Bergen, Bergen, Norway; 5 Department of Otolaryngology, Röpcke-Zweers Hospital, Hardenberg, The Netherlands; 6 Department of Clinical Science, Section for Pediatrics, University Bergen, Bergen, Norway

**Keywords:** Larynx, Exercise capacity, Exercise-induced asthma, Exercise-induced laryngeal obstruction, Vocal cord dysfunction, Exercise testing, Respiratory measurement

## Abstract

Exercise-induced shortness of breath is not uncommon in otherwise healthy young people. Based on the presenting symptoms alone, it is challenging to distinguish exercise-induced asthma (EIA) from exercise-induced obstruction of central airways, sometimes leading to diagnostic errors and inadequate treatment. Central airway obstruction usually presents with exercise-induced inspiratory symptoms (EIIS) during ongoing exercise. EIIS tends to peak towards the end of an exercise session or immediately after its completion, contradicting symptoms of EIA typically peaking 3–15 min after the exercise has stopped. EIIS is usually associated with some form of laryngeal obstruction. Transnasal flexible laryngoscopy performed continuously throughout an incremental exercise test from rest to exhaustion or to intolerable symptoms is usually diagnostic, and also provides information that is important for further handling and treatment. Reflecting the complex anatomy and functional features of the larynx, exercise-induced laryngeal obstruction (EILO) appears to be a heterogeneous condition. Contradicting previous beliefs, recent literature suggests that laryngeal adduction in a majority of cases starts in supraglottic structures and that vocal cord adduction (VCD) most often occurs as a secondary phenomenon. However, EILO is poorly understood and more and better research is needed to unravel causal mechanisms. The evidence base for treatment of EILO is weak. Speech therapy, psychotherapy, biofeedback, muscle training, anticholinergic aerosols have all been applied, as has laser supraglottoplasty. Randomized controlled trials with well-defined and verifiable inclusion and success criteria are required to establish evidence-based treatment schemes.

**Electronic supplementary material:**

The online version of this article (doi:10.1007/s00405-014-3159-3) contains supplementary material, which is available to authorized users.

## Background

Ideally, ventilation should not limit exercise capacity in young and otherwise healthy individuals [1]. However, various forms of airway obstruction do occur, increasing the work of breathing and producing exercise-induced respiratory symptoms. Thus, exercise-induced shortness of breath is not uncommon, and a scenario most physicians must be prepared to encounter. Principally, airway obstruction inside the thoracic cage produces expiratory symptoms (as in asthma), while obstruction outside the thoracic cage produces inspiratory symptoms. The purpose of this article is to provide state-of-the-art knowledge on diagnostics and treatment modalities of central airway obstruction in patients presenting with exercise-induced inspiratory symptoms (EIIS). The article focuses primarily on the role played by the larynx, representing the “entrance valve” and the narrowest passage of the airway tree.

Authors have related EIIS to distinct diagnoses, conditions or dysfunctionalities in specific structures, most often the vocal folds. Hence, the term vocal cord dysfunction (VCD) has become widely used. However, the proposal that the vocal folds are causally involved in EIIS is based on weak evidence and often not verified by objective methods [2, 3]. A plethora of diagnostic terms has been used in the literature to label relatively similar clinical entities, and vice versa, similar labels are given to conditions that may very well represent different diseases [4]. There is no agreement on important issues like diagnostic work-up, etiology, and treatment. This unfortunate situation may be related to heterogeneities within the patient populations, so far not properly acknowledged. Thus, patients presenting symptoms in different situations (e.g., exercise vs. non exercise) have been lumped together in studies, somehow assuming a similar etiology [5]. Moreover, patients referred to third level specialized otorhinolaryngology clinics most certainly differ from patients seen at clinics dealing mainly with respiratory diseases. As research groups will interpret symptoms and findings within the context of their own experience and expertise, there may in fact be no genuine disagreements, only genuine attempts to interpret a heterogeneous reality.

**Fig. 1:**
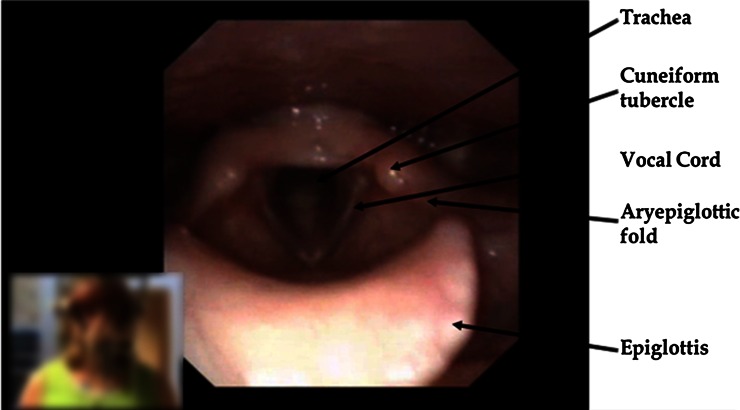
Normal larynx, as observed transnasally in a flexible laryngoscope, with the patient in the *lower left corner* (anonymized), epiglottis (*at front*) the cuneiform tubercles and the aryepiglottic folds represent supraglottic structures. The vocal cords (glottis) and the upper part of the trachea are seen below

In this article, we will use the term exercise-induced laryngeal obstruction (EILO) to describe laryngeal airflow obstruction during exercise in patients with no obvious laryngeal pathology at rest (Fig. 1). By nature, laryngeal obstruction can occur through a reduction in the size of the supraglottic space (supraglottic EILO) by anteromedial rotation of the cuneiform tubercles, medial movements of the aryepiglottic folds or retroreflective positioning or movements of the epiglottis. Laryngeal obstruction can also occur by reduction of the space between the vocal folds (glottic EILO or VCD). Considering the complex nature of the larynx, combinations of these scenarios seem plausible.

## Methods

We searched PubMed for “exercise” combined with: vocal cord dysfunction, paradoxical vocal fold motion, laryngomalacia, laryngeal obstruction, and laryngeal dysfunction. The search was quality checked by scrutinizing the reference lists of included studies. Systematic assessment of relevance, design and quality was complicated by large variations (or lack of statements) regarding diagnostic methods, patient inclusion, evaluation and treatment. Particularly, studies tended to mix patients with exercised-induced symptoms and those with symptoms presenting primarily at rest, two conditions that are likely to represent different disease domains. Stating clearly in the text when doing so, we have allowed ourselves to express personal opinions, based on articles published by our group and a cumulative experience from more than 700 patients with EIIS, examined endoscopically during exercise in the past 15 years. Thus, the article does not fully comply with all requirements for a systematic review, and is biased by the authors’ experience and access to large numbers of high-quality videos of patients presenting with EIIS, suffering from EILO [6].

## Diagnosing EILO

Exercise-induced inspiratory stridor is often confused with symptoms of exercise-induced asthma (EIA) [7, 8]. The prevalence of EIA has been estimated to 8–10 % in unselected childhood populations and approximately 35 % in children with untreated asthma [9]. The incidence of EILO was reported to be as high as 7.5 % in unselected young people in Copenhagen [10]. Treatment with asthma medication is common in patients with EIIS, often with little or no effect [11, 12]. In our own study [13], 85 % of patients with EIIS had received asthma treatment prior to being diagnosed with EILO, with no effect on exercise-related symptoms in 64 %. However, it is important to bear in mind that EIA and EILO may coexist [2, 3, 11, 12, 14].

Three main criteria have been proposed as essential to the diagnosis EILO): (1) Clinical symptoms of EIIS. (2) Confirmatory pulmonary function findings and (3) Laryngeal obstruction, verified laryngoscopically [2, 3, 15–18].

### Clinical symptoms

Patients are often unable to account for their symptoms in detail at first visit, but rather present with some form of respiratory complaints occurring during exercise, often made worse by cold climate and also not responding as expected to asthma medication. Two key questions should be answered regarding symptoms: their position in the respiratory cycle (inspiratory vs. expiratory) and in the exercise session (during or immediately after vs. 10–15 min after). If properly informed, most patients are able to respond to these questions if rescheduled a week or two later.

In a test situation, EIA is characterized by changes in forced expiratory volume in first second (FEV_1_) occurring from before vs. after the exercise session [19]. The symptoms occur as a response to exercise; i.e., slowly evolving during the first 3–15 min after stopping and mainly involving the expiratory part of the respiratory cycle [20]. A correctly performed test for exercise-induced asthma is of diagnostic value also with respect to EIIS, as inspiratory symptoms should be revealed by the high-intensity exercise—if present. Breath patterns and symptoms during the test must be recorded, not only changes in FEV_1_ after exercise. EIIS is usually characterized by a fairly typical pattern, starting with increasing breathing difficulties accompanied by a prolonged inspirium with course or high-pitched or stridor-like inspiratory breaths sounds, sometimes progressing to clear-cut stridor, hyperventilation attacks or frank panic reactions, evolving in parallel to the increasing ventilatory requirements [15, 21]. Pain reactions located to the chest or throat area are relatively common (unpublished own data). It is important in this context to remember that EIIS is not a well-defined entity, but rather a series of symptoms that usually occur in sequence, somewhat open to subjective interpretation. A thorough description of observations made during an exercise test should serve to differentiate symptoms of asthma from EIIS.

### Pulmonary function tests

Several studies have described abnormal resting flow-volume loops (FVL) in patients with EIIS [2, 3, 7, 22]. According to Christopher [15] the most common cause of blunted or truncated inspiratory FVL is inadequate instruction, suboptimal effort or inability to perform the procedure. The repeatability of the findings is poor [23, 24] and the sensitivity regarding identification of patients with EIIS is low [2, 3, 25, 26]. Various cut-off levels for inspiratory vs. expiratory flow ratios have been suggested, with no validated consensus obtained [3, 22, 25, 27].

In summary, there is currently no evidence to suggest that EILO can be confirmed or rejected by resting pulmonary function tests (PFT) alone, and to use exclusively FVLs to set specific intrinsic laryngeal diagnoses such as VCD seems futile. Nevertheless, PFTs are important, partly to distinguish asthma from EIIS but also to indicate the presence of structural central airway obstructions such as subglottic stenosis, laryngo-tracheo-bronchomalacia or intrathoracic compressions of various forms. Distinct and reproducible flatting of the inspiratory and/or expiratory parts of the FVL in patients with EIIS should incite further assessment [15].

### Laryngoscopic visualization

Visualization of laryngeal structures during exercise has been proposed the “gold standard” for diagnosing EILO [2, 3, 15, 17, 18, 28–30]. Christopher showed in a review of 355 articles on VCD that laryngoscopy during ongoing symptoms had not been performed in 38 % of patients [15]. Newman wrote that laryngoscopy was diagnostic in only 60 % of symptomatic patients [12]. There are several possible explanations for this lack of findings. Importantly, symptoms resembling EIIS may be unrelated to larynx in an unknown proportion of patients, since obstruction of trachea or main bronchi can produce symptoms difficult to distinguish from those of EILO [13]. Second, in most studies laryngoscopies were performed post-exercise, which is a questionable method. EIIS usually peaks at maximum ventilation and often resolves rapidly thereafter. As the minute ventilation falls rapidly after stopping of exercise [1], the time required to introduce a laryngoscope in a distressed patient may result in false-negative tests. Alternatively, the test situation may be inadequate in that patients are unable to exercise to the level necessary to reproduce their symptoms. A final issue is lack of consensus regarding what are normal and what are abnormal findings.

### Continuous laryngoscopy exercise test (CLE test)

**Fig. 2:**
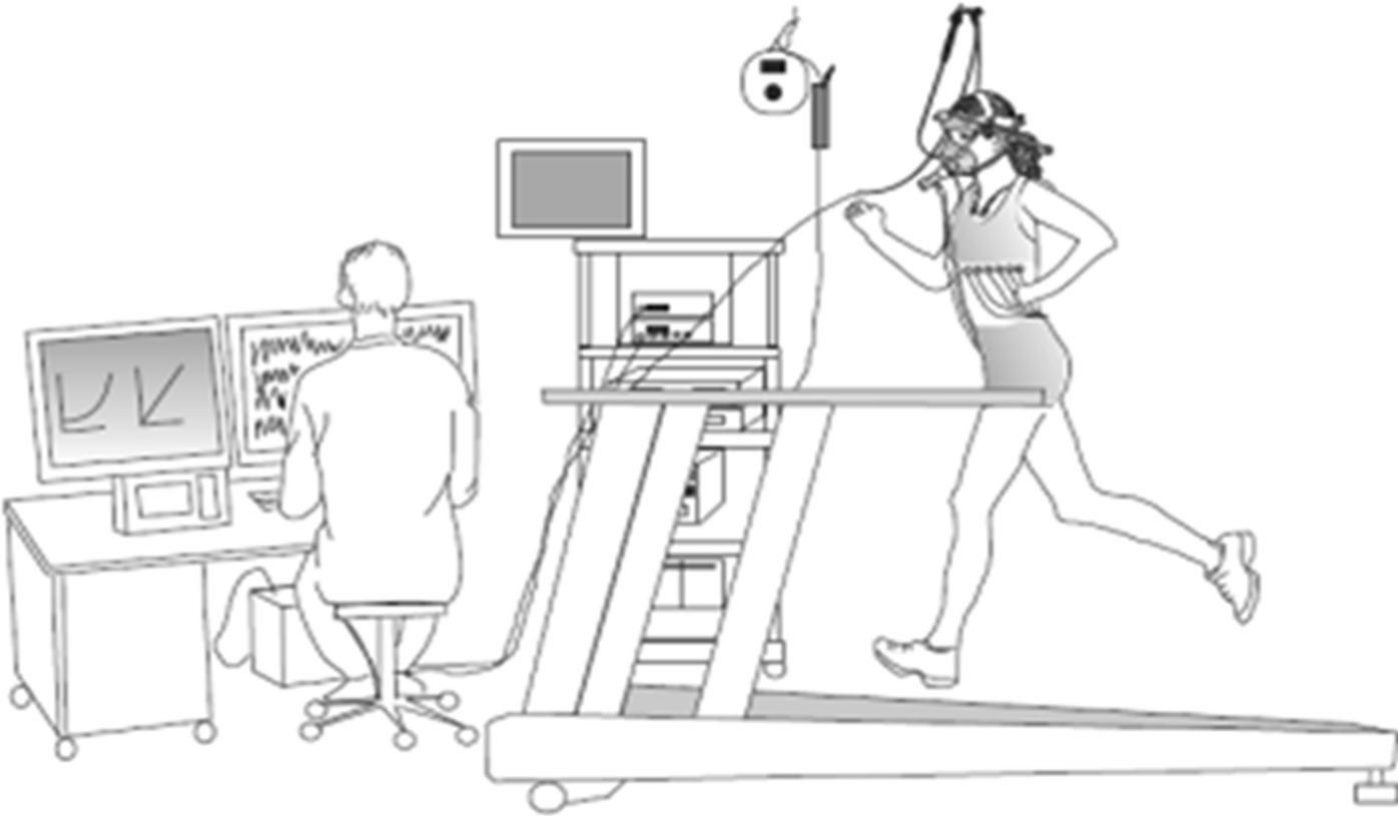
Continuous laryngoscopy exercise test (CLE test). Illustration: GØrill Skaale Johansen

We have published a method for continuous monitoring of the larynx throughout ongoing maximal cardiopulmonary treadmill exercise; i.e., continuous laryngoscopy exercise test (CLE test) (Fig. 2) [21]. The method is easy to perform and well tolerated from the age of 5 years. It allows for documentation of visible alterations and movements in laryngeal structures during all phases of the respiratory cycle throughout a complete exercise test. Synchronized cardiopulmonary exercise data and video recordings of the upper part of the body and sound tracks are stored for later review and analysis [21]. It has been argued that the CLE test is too resource intensive, and that a laryngoscope held by the hand with the patient exercising on a bike will serve the same purpose. This may be so, but the laryngoscope must be in place before onset of symptoms and throughout the full length of the exercise session. Only then can important characteristics be revealed and documented, such as which structures incited and perpetuated the events.

### Mode of exercise used to reproduce EIIS

Treadmill running, ergometer cycling or stair climbing have all been used to provoke and reproduce symptoms of EIIS [3, 17, 29, 31]. Ideally, the mode of exercise should be tailored to the individual patient, based on triggers identified from the medical history. In a laboratory setting, one must compromise. More important than the mode of exercise is to ensure that the exercise continues to exhaustion or intolerable symptoms. In most young people and fit adults, treadmill exercise is better than bicycle to achieve this aim [32]. Tervonen used a stationary bicycle in their study and was unable to reproduce symptoms in 50 % of patients and made laryngeal findings in only 30 % [17], demonstrating the importance of this issue.

### Normal laryngeal movements during exercise

The human larynx protects the lower airways from aspiration, facilitates respiration and plays a key role in phonation. The supraglottic part, i.e., aryepiglottic fold, cuneiform tubercles and epiglottis, are supported by muscles, ligaments and membranes. Tiny fibers from the lateral belly of vocal fold muscles are stretched cranially into the aryepiglottic fold. During exercise, the larynx normally opens fully and the epiglottis rotates anteriorly towards the base of the tongue [29], stretching the aryepiglottic folds, thereby allowing for increased airflow with the least possible increase of airflow resistance [28]. Several muscles are active in this process [33]. The motion of the arytenoids occurs in three dimensions, i.e., sliding, tilting and rotation along the vertical axis. The top of each cartilage determines the position of the dorsal end of the aryepiglottic folds, whereas the anteriorly and caudally placed vocal processes determine the positions of the dorsal end of the membranous part of each vocal fold.

In the abducted position, the dorsal part of vocal folds is lifted cranially. Most of the airflow through larynx takes place in the dorsal part of the glottic aperture. The posterior cricoid muscle (PCA) acts as an abductor of the glottis; its activity synchronized and in a phasic interaction with the diaphragm muscle [33–35].

A slight adduction of the aryepiglottic folds at maximum minute ventilation was observed in 40 % of subjects without respiratory complaints participating in a study performed by our group [13] and, therefore, considered to be a normal phenomenon [36]. Bent and co-workers [29] made similar observations. Regarding the vocal gap, McFadden et al. [2] proposed that an adduction exceeding 50 % was consistent with VCD. These issues need to be addressed in larger studies. Particularly, we have no knowledge on what are normal or optimal relations between body size, ventilatory requirements and the size of the laryngeal aperture. Thus, a similar extent of adduction is likely to have different consequences in a narrow compared to a wide larynx and also for a competing athlete compared to a sedentary person.

### Diversity of findings in patients with EIIS

In a study of 151 patients with EIIS using the CLE test, we observed a variety of response patterns, but the typical patient was characterized by a normal larynx at rest and moderate to severe adduction of laryngeal structures during exercise [13]. Supraglottic anteromedial motion of the aryepiglottic folds and of the cuneiform tubercles was involved in the majority of patients, and was most often the inciting event. If present, vocal fold adduction usually occurred as a secondary event or in parallel to supraglottic adduction. In some patients, a dorsal flexion of the epiglottis seemed to represent the air flow obstacle, seemingly as a primary event. Clinically, EIIS evolved in parallel to gradually increasing laryngeal obstruction. Clear-cut stridor appeared to be related to adduction of the vocal folds, and panic reactions to severe vocal fold adduction. Only four (3 %) of 151 patients had a primary glottic adduction or VCD, as defined by Christopher in 1983 [22]. Nine (6 %) subjects had a normal laryngeal response to exercise, but symptoms resembling EIIS; i.e., EIIS but no EILO. Further investigations revealed structural obstruction of central airways in seven, caused by vascular compression, tracheomalacia and subglottic stenosis. This heterogeneity of findings has been confirmed also in other and smaller studies that utilize laryngoscopy during exercise to investigate patients complaining of EIIS [10, 17, 37, 38].

After hundreds of CLE tests performed in patients with EIIS (5–65 years of age), we are unable to link EIIS to one single anatomical structure or causal factor, and certainly not exclusively to the vocal folds. Adduction of the vocal folds is not the primary event in most patients, but rather a consequence or an associated phenomenon secondary to supraglottic alterations. These findings are supported by other studies, utilizing laryngoscopy as diagnostic method [10, 39]. The anatomy, physiology, nervous regulation and function of the larynx are complex, and heterogeneities regarding exercise-induced malfunction, therefore, plausible and not surprising.

It has recently been argued that VCD may be diagnosed based on the presenting symptoms alone, and that laryngoscopy is difficult and even unnecessary [5]. This strongly contradicts the experience gathered by our group and by others, with EIIS being associated with a spectrum of structural and functional abnormalities [10, 13, 37, 38]. These distinctions are of practical importance, as patients with severe collapse of supraglottic structures benefit from laser supraglottoplasty [40, 41]. Moreover, this attitude may leave patients with EIIS due to extra-laryngeal obstruction with no (or incorrect) diagnosis.

## Potential casual aspects of EILO

### Aerodynamic principles

The Bernoulli principle states that increasing airflow through a tube creates increasing negative pressures within the tube [42]. Depending on airflow velocity and turbulence and the strength of the supporting structures, sooner or later the tube will yield to these forces. At what flow-rates laryngeal structures will yield is determined by the area and configuration of the laryngeal opening, “internal laryngeal solidity” and support from surrounding structures. Thus, EILO may be explained by poor support from muscles, ligaments or laryngeal cartilages.

A weakness of the PCA muscle or of the structures that stabilize the arytenoids and keep them upright and laterally positioned may be involved [35]. This will reduce the size of the laryngeal aperture, possibly to below a critical level required for laminar and advantageous aerodynamic inspiratory flow [43]. A secondary medial motion of the vocal folds may be explained by an increased negative pressure in the space between the vocal folds, due to changes of airflow induced by medial movements of the structures above [43]. This sequence would fit the observations made in most participants of our studies. It has been speculated whether there is a connection between infantile laryngomalacia and subsequent EILO. The evidence for this is relatively modest [44].

### Laryngeal hyperreactivity and changes in reflex interaction

Reflexes are important for laryngeal function in relation to respiration, swallowing and protection against aspiration. The idea of “reflex associated VCD” is that direct stimulation of sensory nerve endings in the respiratory tract may induce protective reflex loops, leading to laryngeal closure [45]. Mechanical or chemical stimulation of the supraglottic mucosa or direct stimulation of the superior laryngeal nerve may activate the laryngeal adductor reflex to protect the airway from aspiration or asphyxiation [46]. A variable sensitivity and intensity of this reflex interaction could conceivably lead to a corresponding variability in the threshold for laryngeal closure. Conditions such as allergy, reflux and infections may influence these reflex interactions. So far, the evidence supporting these mechanisms is weak.

### Laryngopharyngeal reflux (LPR)

Gastroesophageal reflux disease (GERD) has been associated with VCD by several authors [27, 47]. The argument has been that acidic reflux reaching the laryngopharyngeal area should induce a hyperexcitable state [27, 48]. If a causal relationship is present, one would expect that treatment with proton-pump inhibitors (PPIs) should reduce reflux symptoms as well as EIIS. In a study by Maturo et al., three patients with VCD and LPR were treated with PPI with a positive effect on LPR, but not on VCD [5]. A recent study indicated that subjects with high reflux symptom index in fact had reduced laryngeal sensitivity [49]. It is important to remember that the prevalence of GERD in unselected populations may be as high as 10–60 % [50]. Causal relationships involving conditions with this kind of prevalence should be proposed with caution.

### Psychological aspects and EILO

VCD tend to be interpreted within a psychological paradigm. In their review article, Leo and Konakanchi [51] reported from a sample of 171 cases with paradoxical vocal cord motion (PVCM), and found that only 7 % did not have a psychiatric diagnosis. Others have claimed that VCD is associated with conversion disorders, representing the physical manifestation of underlying psychological problems [52]. Sexual abuse in early childhood is still being put forward, based on a small study from the 1990s [53].

In this context, it is probably important to distinguish laryngeal obstruction occurring at rest from that induced by exercise. Based on decades of personal experience working with children and adolescents with exercise-induced symptoms, we have found no reason to suspect that a majority of patients with EIIS and EILO suffer from mental disorders. It is our impression that most are otherwise healthy and physically active young people who benefit from being explained that their breathing problem is not dangerous, and that there is nothing mentally wrong with them. However, we have certainly observed that many patients are concerned and sometimes frightened by their symptoms, and therefore reluctant to expose themselves to situations they know will provoke them. In our opinion, this reaction is understandable, considering the trauma of breathlessness during heavy exercise. The panic reactions observed in some individuals should not be interpreted within a psychiatric context, but rather as a response to the choking feeling of laryngeal collapse. Post-exercise introduction of a laryngoscope in a distressed patient may simply reveal adducted vocal folds and panic, and therefore erroneously be interpreted as paradoxical vocal fold motion induced by hysteria. Thus, post-exercise laryngoscopy entails a high risk of making the classical mistake of reverse causality; the panic reaction does not cause EILO, but is caused by EILO.

As this group of patients is characterized by its heterogeneity, we do not exclude that stress, anxiety and competitive personalities may worsen and possibly also trigger symptoms and findings, but argue that an organic substrate makes the EILO response possible in most patients. This point of view is strengthened by the convincing effect of surgical treatment in selected severe cases [41].

### Environmental conditions and EILO

In exercise-induced asthma, temperature and humidity are important pathogenetic factors [54, 55]. Regarding EIIS and EILO little is known. In our experience, performers of particularly winter sports, but also swimmers, handball, basket and soccer players seem to be over-represented. Most patients express that a cold climate reduces their tolerance to exercise. These findings correspond partly to the descriptions given by Rundell, reporting that inspiratory stridor was more prevalent in outdoor (8.3 %) compared to indoor athletes (2.5 %) [3], indicating that environmental factors are implicated in the pathogenesis of EILO as of EIA.

### Age, gender and physical capacity in relation to EILO

EIIS seems to start in early adolescence in a majority. The design of the aryepiglottic folds and the cuneiform tubercles make the supraglottic opening relatively narrower in adolescents than in adults and the epiglottis is longer and may be curved or omega shaped [56, 57]. Relative to body size, maximum oxygen uptake peaks in adolescence, necessarily also reflected by the maximum minute ventilation. These factors may all contribute to the age distribution of EILO. Most agree that EILO is more common in girls [15, 58, 59]. Anatomical studies have shown no gender differences in the relative size of the laryngeal aperture before puberty, while there are significant gender differences throughout the pubertal growth spurt [57, 60].

## Prognosis of EILO

It has been suggested that laryngeal growth and maturation during puberty should make the larynx more robust to inward forces at high minute ventilation [61]. In a 2- to 5-year follow-up study, Maat et al. [41] were unable to show that growth by itself could cure EILO. Patients reported less symptoms but also lower levels of activity, suggesting changes to a lifestyle not challenged by laryngeal airflow limitations. Most patients reported that to be assigned a diagnosis and to actually see what took place in their larynx was important for a perception of safety in relation to exercise and to maintain a reasonable level of physical activity. Among those who had been treated surgically, nearly all were entirely cured [41].

## Treatment of EILO

Inclusion to studies aiming to address treatment of EILO (or exercise-induced VCD) was, in the majority of articles, based on symptoms presented by the patients. The same applied to outcome measures; i.e., in most studies inclusion as well as success rates was based on subjective patient reports. Moreover, in most studies patients with EIIS were treated as if they were all suffering from one defined disease entity, often labeled exercise-induced VCD. Few studies have based inclusion and treatment strategies on verifiable findings or created a targeted strategy to deal with this. Given the heterogeneity of laryngeal findings that has been reported in patients with relatively similar symptoms [13, 18, 39], it seems that conclusions should be interpreted very cautiously. Another complicating factor is the role played by placebo effects in studies utilizing open label designs and methods such as psychotherapy or speech therapy [2, 5, 7, 11, 15, 26, 62–65]. Inhaled ipratropium bromide applied locally prior to activity has been reported to prevent exercise-induced VCD [66]. Different forms of biofeedback techniques have been proposed [67], as has inspiratory muscle training (IMT) [68–71]. Surgical supraglottoplasty has been used to treat patients with severe supraglottic EILO and positive effects have been reported by several [29, 37, 39, 40, 72–74]. Selection of patients for surgical treatment should be performed with great care, particularly avoiding those with a primary glottic EILO [40, 41] and potential gains must be weighed against the risk of potential complications. The place for surgery in the treatment of EILO has not been settled.

Video-recorded verification of laryngeal obstruction may be of value not only as a diagnostic tool, but also as a therapeutic measure. Simply observing their own malfunctioning larynx is of help in a majority of patients with mild or moderate disease. The video recordings are also highly educational in the process of providing advice regarding what is a rational respiratory pattern during exercise. However, these are clinical observations, not substantiated scientifically. In conclusion, there is a need for randomized controlled trials with inclusion as well as outcome assessment performed with objective measures.

## Concluding remarks

Obstructions of central airways are important causes of exercise-induced inspiratory symptoms (EIIS) in young and otherwise healthy individuals. This is a large, heterogeneous and vastly understudied group of patients. The symptoms are too often confused with those of asthma. Laryngoscopy performed as symptoms evolve during increasing exercise is pivotal, since larynx plays an important role in a majority. Abnormalities vary between patients, and laryngoscopic findings are of value for treatment and further handling. Causal mechanisms are generally poorly understood. The evidence base for most treatment options is weak, but most patients seem to benefit from individualized information and guidance. Surgical treatment may be indicated in well-defined and severe cases. A systematized clinical approach, more and better research utilizing objective methodology as well as randomized controlled treatment trials are of utmost importance in this field of respiratory medicine.

## Electronic supplementary material

Below is the link to the electronic supplementary material.

Supplementary material 1 (WMV 2434 kb)

Supplementary material 2 (WMV 7379 kb)

Supplementary material 3 (WMV 24146 kb)

## Abbreviations

CLE test: Continuous laryngoscopy exercise test
EIA: Exercise-induced asthma
EIIS: Exercise-induced inspiratory symptoms
EILO: Exercise-induced laryngeal obstruction
FEF_50_/FIF_50_: The ratio between forced expiratory flow at 50 % of forced expiratory vital capacity and the forced inspiratory flow at 50 % of forced inspiratory vital capacity
FEV_1_: Forced expiratory flow in the first second
FVL: Flow volume loop
GERD: Gastroesophageal reflux disease
IMT: Inspiratory muscle strength training
LPR: Laryngopharyngeal reflux
MIF_50_/MEF_50_: The ratio between maximal inspiratory flow at 50 % of forced inspiratory vital capacity and the maximal expiratory flow at 50 % of forced expiratory vital capacity
PCA-muscle: Posterior cricoarytenoideus muscle
PFT: Pulmonary function test
PPI: Proton pump inhibitor
PVCM: Paradoxical vocal cord motion
RSI: Reflux symptom index
VCD: Vocal cord dysfunction
